# Supplementary material for: YAP and TAZ are transcriptional co-activators of AP-1 proteins and STAT3 during breast cellular transformation
Source: eLife. 2021 Aug 31;10:e67312. doi: 10.7554/eLife.67312 (PMC8463077; doi:10.7554/eLife.67312)
Supplement: Supplementary file 1. [file elife-67312-supp1.pdf]

Table S1. Correlation between (A) biological replicates and (B) number of binding sites of the indicated proteins in ER-Src (ethanol or tamoxifen; wild-type and YAP or TAZ knockout (KO) and MDA-MB-231 cells.

A. ChIP-seq signal correlation coefficient between replicates

**TF**	YAP	TAZ	TEAD	STAT3	JUNB
**ER-Src; ETH**	0.705	0.763	0.779	0.890	0.918
**ER-Src; TAM**	0.683	0.855	0.772	0.950	0.912
**MDA-MB-231**	0.908	0.886	0.878	0.668	0.915

B. ChIP-seq IDR peak numbers

	YAP	TAZ	TEAD	STAT3	JUNB
**ER-Src; ETH**	25,975	32,587	15,486	3,680	37,886
**ER-Src; TAM**	22,509	37,888	19,467	18,243	44,952
**MDA-MB-231**	43,387	32,820	15,870	6,316	59,271

	YAP in TAZ KO	TAZ in YAP KO
**ER-Src; ETH**	29,335	49,118
**ER-Src; TAM**	39,230	35,221
